# The Muscle-Liver Immunometabolic Axis in Liver Transplantation: A Decisive Role in Outcomes and Mechanisms

**DOI:** 10.7150/ijbs.132050

**Published:** 2026-06-25

**Authors:** Zhu Li, Jinyan Chen, Huigang Li, Yuhang Li, Peiru Zhang, Yiyu Chen, Rongsen Wang, Ke Yang, Ximing Wang, Xinjun Zhuang, Yiming Su, Jinxin Xu, Shusen Zheng, Xiao Xu, Di Lu

**Affiliations:** 1 School of Basic Medical Sciences and Forensic Medicine, Hangzhou Medical College, Hangzhou 310000, China.; 2 Institute of Translational Medicine, Zhejiang University School of Medicine, Hangzhou 310000, China.; 3 Zhejiang University School of Medicine, Hangzhou 310000, China.; 4 Zhejiang Chinese Medical University, Hangzhou 310000, China.; 5 Hangzhou Medical College, Hangzhou 310000, China.; 6 NHC Key Laboratory of Combined Multi-organ Transplantation, Hangzhou 310000, China.; 7 General Surgery, Cancer Center, Department of Hepatobiliary & Pancreatic Surgery and Minimally Invasive Surgery, Zhejiang Provincial People's Hospital (Affiliated People's Hospital), Hangzhou Medical College, Hangzhou 310000, Zhejiang, China.; 8 Hepatobiliary Center and Transplantation Center, The First Affiliated Hospital with Nanjing Medical University, Nanjing 210000, Jiangsu, China.; 9 Li Huili Hospital Affiliated to Ningbo University, Ningbo 315000, Zhejiang Province, China.

**Keywords:** liver transplantation, muscle-liver axis, sarcopenia, frailty, immunosuppression, immunometabolism

## Abstract

Liver transplantation has transformed survival for end-stage liver disease, shifting clinical attention from acute rejection to longer-term vulnerability driven by metabolic dysfunction and loss of skeletal-muscle reserve. In this Review, we examine liver transplantation through the muscle-liver immunometabolic axis across the waitlist, perioperative and follow-up phases. Clinical evidence consistently links sarcopenia, frailty, myosteatosis and sarcopenic obesity with infection, prolonged hospitalization and excess mortality after transplantation, while muscle quantity, quality and function often diverge, exposing the limitations of weight-based assessment alone. Mechanistically, nutrient sensing, inflammatory signaling and immune competence converge through pathways involving AMPK, mTOR, PGC-1α, myokines, hyperammonemia and immunosuppressant exposure. These interactions help explain why indispensable immunosuppression may simultaneously protect the graft and impair metabolic recovery. We highlight muscle-directed strategies — including structured prehabilitation and rehabilitation, protein and branched-chain amino acid support, ammonia-aware interpretation and selected anabolic approaches — as pragmatic entry points for improving resilience. However, translation remains limited by heterogeneous definitions, sparse longitudinal phenotyping and a lack of transplant-specific randomized evidence.

## Introduction

**Figure 1:**
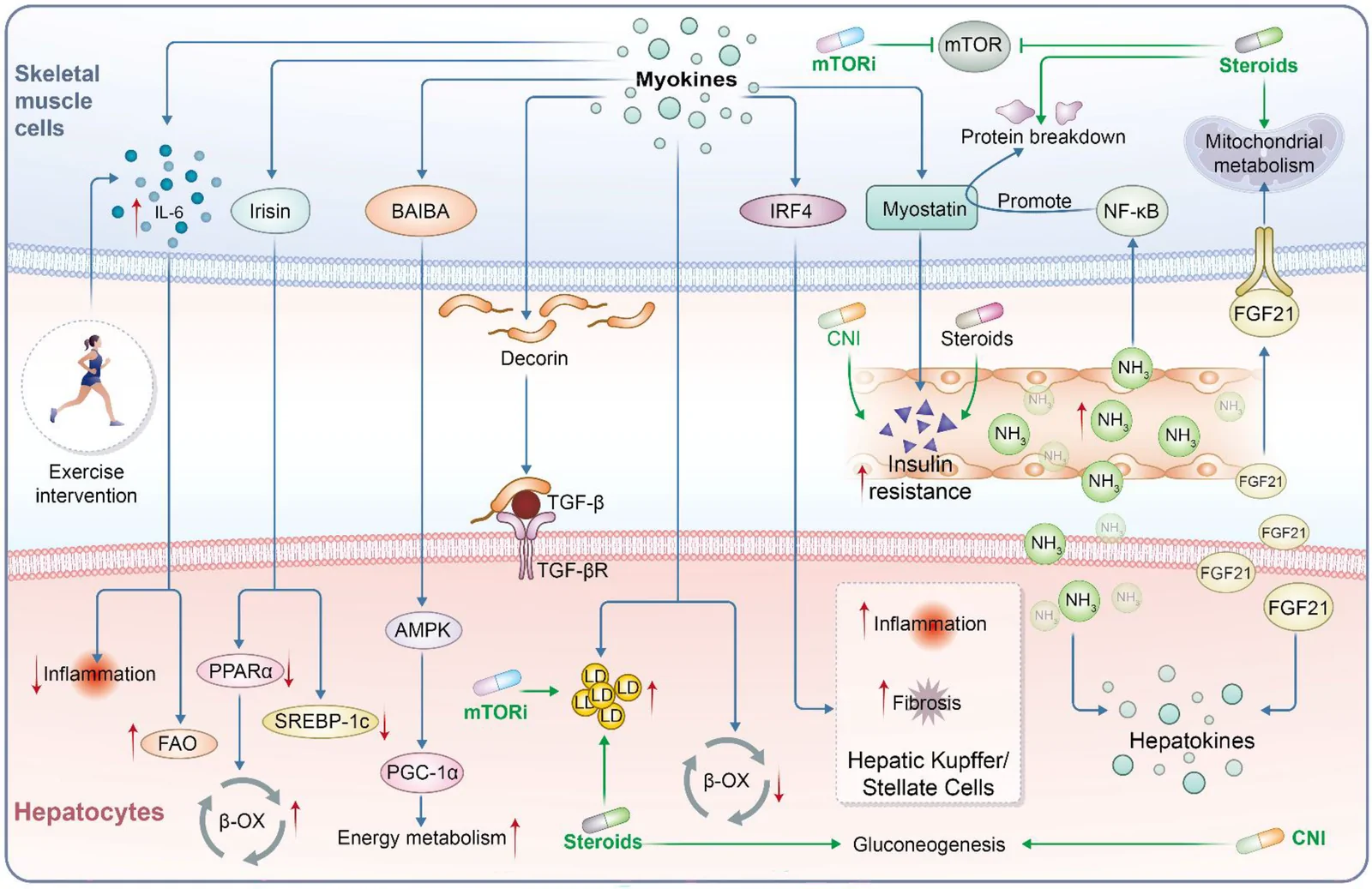
** Exercise and immunosuppression regulate the post-transplant muscle-liver immunometabolic axis.** Schematic illustration of bidirectional immunometabolic crosstalk between skeletal muscle and liver after liver transplantation. Exercise is associated with IL-6, irisin, BAIBA, and decorin signaling and may modulate IRF4/FSTL1-related pathways, and is associated with reduced inflammation, modulation of TGF-β signaling, activation of AMPK-PGC-1α-related pathways, enhanced FAO and β-OX, improved energy metabolism, and reduced hepatic lipogenesis. In contrast, post-transplant immunosuppressive agents, including mTORi, corticosteroids, and CNIs, impair mTOR-dependent anabolic signaling, mitochondrial metabolism, and insulin responsiveness, thereby promoting protein breakdown, insulin resistance, gluconeogenesis, lipid dysregulation, ammonia-associated inflammatory stress, NF-κB activation, myostatin-related catabolic signaling, and inflammatory and fibrogenic responses in hepatic Kupffer and stellate cells. FGF21 represents a hepatokine-mediated feedback signal from liver to skeletal muscle. Red arrows indicate increased activity or expression; green inhibitory lines indicate suppression.

**Figure 2:**
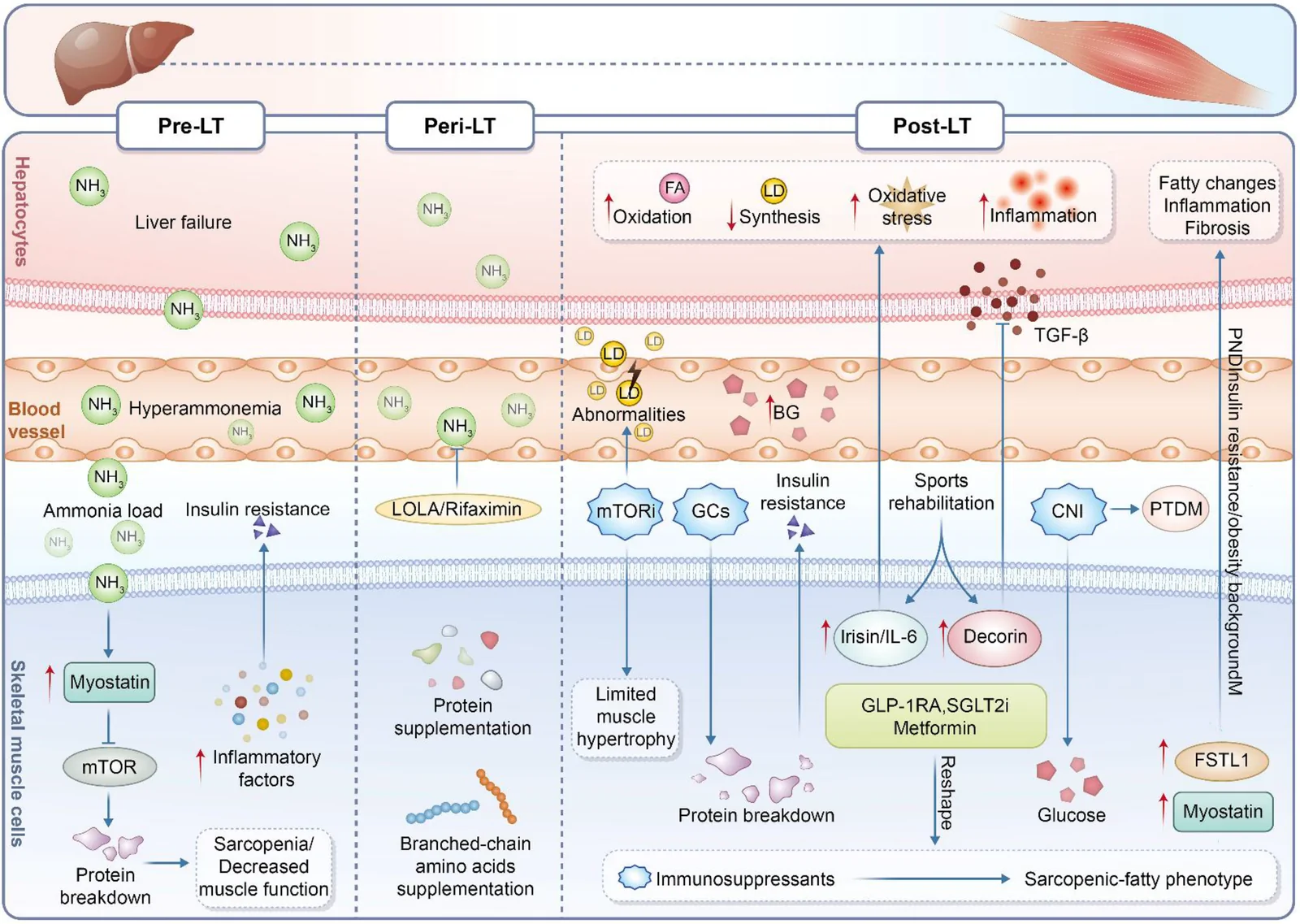
** Stage-specific remodeling of the muscle-liver axis throughout the liver transplantation continuum.** During the pre-liver transplantation stage, liver failure and hyperammonemia promote ammonia overload, insulin resistance, inflammation, protein breakdown, and sarcopenia. During the peri-liver transplantation stage, metabolic abnormalities and immunosuppressive exposure limit muscle recovery, although ammonia-lowering therapy and nutritional supplementation may offer partial support. During the post-liver transplantation stage, persistent metabolic stress and immunosuppressive treatment contribute to oxidative stress, inflammation, fibrosis, post-transplant diabetes mellitus, and the progression toward a sarcopenic-fatty phenotype. Exercise rehabilitation, metabolic therapy, and muscle-derived factors may help modulate the muscle-liver axis after transplantation.

Liver transplantation (LT) is a life-saving therapy for end-stage liver disease, and advances in surgical technique and immunosuppressive regimens have markedly improved short-term survival 1. As a result, the clinical focus has increasingly shifted from acute rejection to longer-term complications. This shift reframes LT care from “preventing rejection” to “managing long-term vulnerability”, where immunologic control is necessary but rarely sufficient for durable recovery. Metabolic sequelae—including new-onset diabetes, hyperammonemia, obesity and progressive loss of skeletal muscle—are now common in LT recipients and are linked to adverse graft and patient outcomes 2-5. Skeletal muscle is increasingly recognized as a central node in this immunometabolic landscape, integrating nutrient sensing, inflammation and immune competence 6-9. Many LT candidates present with frailty and/or sarcopenia (loss of muscle mass, strength and/or quality), which is consistently associated with worse pre- and post-transplant trajectories. Clinically, frailty and sarcopenia should be treated as actionable markers of limited reserve that warrant structured assessment and escalation of supportive care, rather than as descriptive comorbidities. After LT, patients with sarcopenia or incomplete muscle recovery experience higher rates of infection, longer hospital stays and reduced survival. Taken together, these observations imply that muscle status remains a determinant of post-LT vulnerability even when hepatic function is restored. Conversely, preserving or restoring muscle may represent a clinically modifiable target within LT care pathways and may help improve immunometabolic resilience across the waitlist, perioperative, and follow-up phases. The principal pathways through which exercise and immunosuppressive exposure reshape post-transplant muscle-liver immunometabolic signaling are summarized in Figure 1. The dynamic remodelling of the liver-muscle axis across the waitlist, perioperative and follow-up phases is summarized in Figure 2. However, the current evidence base is dispersed across outcome cohorts, mechanistic studies and intervention reports, with limited standardization in definitions and endpoints—hindering translation into a reproducible clinical framework. This Review synthesizes clinical and mechanistic evidence on the muscle-liver axis in transplantation, focusing on how muscle status and muscle-derived signals shape metabolic and immune pathways relevant to LT 7, 10. We first summarize the clinical immunometabolic challenges in LT, then examine muscle-liver crosstalk, including myokines, metabolic regulators and immune mediators, and how immunosuppressive therapy may modulate these interactions 8, 10, 11. Finally, we discuss therapeutic implications—spanning exercise, nutrition and emerging pharmacological strategies aimed at leveraging muscle as a modifiable target to improve transplant outcomes 12, 13.

## Search Strategy and Selection Criteria

Two independent researchers (LZ and CJY) searched PubMed and Web of Science Core Collection from database inception to 2 April 2026. No language restrictions were applied. The detailed search strategies for each database are provided in Supplementary Table S1. Eligible articles included clinical, translational, or mechanistic studies involving liver transplant candidates, recipients, or patients with advanced liver disease that were directly relevant to the liver transplant setting and addressed muscle-related phenotypes, the muscle-liver immunometabolic axis, post-transplant complications, immunosuppression-related effects, or related therapeutic strategies. Case reports, conference abstracts, editorials, letters, and studies not directly relevant to liver transplantation were excluded. Titles and abstracts were screened first, followed by full-text assessment of potentially relevant articles. Two independent researchers (LZ and CJY) performed the study selection, and disagreements were resolved through discussion.

## Liver Transplantation and Immunometabolic Challenges

### Sarcopenia, frailty and adverse body composition phenotypes

In parallel with metabolic syndrome, sarcopenia (low skeletal muscle mass with impaired function or low muscle quality) is now recognized as a key predictor of outcomes in cirrhosis and LT 14. In the LT setting, sarcopenia and frailty should be interpreted as “limited physiological reserve” phenotypes that directly condition infection vulnerability, stress tolerance and recovery kinetics, rather than as descriptive comorbidities. Operationally, the clinical signal strengthens when muscle quantity, muscle quality (e.g., radiodensity/myosteatosis) and function are explicitly separated, because these domains can diverge and should not be treated as interchangeable readouts 15, 16. Estimates vary by definition and modality, but 40-70% of patients with cirrhosis awaiting LT are reported to have sarcopenia. This apparent range is not merely epidemiologic noise: in a large systematic review/meta-analysis of pre-LT sarcopenia, reported prevalence spanned 14.7% to 88.3% across cohorts, reflecting heterogeneity in measurement (e.g., SMI vs psoas-based metrics) and cut-offs rather than a true biological contradiction. This may reflect chronic malnutrition, hyperammonemia, inflammation, and physical inactivity in advanced liver disease 15-19. Clinically, low muscle mass and frailty correlate with lower waitlist survival and a higher risk of dropout or delisting due to clinical deterioration 15, 20. Even after transplantation, pre-existing sarcopenia independently predicts postoperative complications. Notably, in the same meta-analysis (30 cohorts; 5,875 recipients), pre-LT sarcopenia was associated with higher post-LT mortality (RR 1.84, 95% CI 1.41-2.39) and with increased risks of post-LT infections, surgical complications, and prolonged ICU length of stay—supporting sarcopenia as a clinically meaningful risk amplifier rather than a surrogate marker. It is also associated with infections, longer ICU and hospital stays, and lower 1- and 3-year survival 21-23. Importantly, sarcopenia often does not fully reverse after LT. Many patients remain sarcopenic or experience further muscle loss during follow-up 22, 24, 25. This persistent signal is partly explained by “quality-first” deterioration: in a CT-based cohort, low pre-LT muscle density (a myosteatosis proxy) was linked to markedly worse survival (e.g., 1-year survival 63% vs 90%) and an approximately threefold higher adjusted hazard of death (HR 2.985), while low muscle mass alone showed weaker associations—highlighting why muscle quality deserves independent emphasis in LT risk models 26. Potential contributors include cumulative corticosteroid exposure. Post-transplant metabolic derangements may also contribute. Deconditioning during recovery is another plausible factor. Direct myotoxic effects of certain immunosuppressants may play a role and are discussed later 27. Taken together, these mechanisms imply that post-LT muscle trajectories are iatrogenically modulated and therefore potentially preventable, provided that monitoring and intervention are embedded as protocolized components of follow-up rather than left to ad hoc rehabilitation. Adverse phenotypes include sarcopenic obesity (low muscle mass with excess adiposity) and myosteatosis (low muscle quality). These phenotypes have been associated with graft loss and mortality 28, 29. Importantly, sarcopenic obesity carries quantifiable excess risk: a 2024 systematic review/meta-analysis reported higher mortality in sarcopenic obesity, including a pooled univariate HR 1.76 (95% CI 1.33-2.33) and a pooled multivariable HR 2.33 (95% CI 1.34-4.04), indicating prognostic impact beyond confounding by baseline severity. Thus, managing body composition, rather than body weight alone, is a central challenge in the post-transplant population 30, 31. This highlights an important limitation of BMI: body weight can normalize or increase while muscle quality deteriorates, so reliance on weight-centric follow-up risks missing the very phenotype most tightly linked to infection and survival signals in several cohorts 26. These observations argue for integrating body composition into post-transplant risk assessment and immunosuppressive decision-making. Clinically, these findings support treating adverse body-composition phenotypes as triggers for intensified nutritional, exercise, and surveillance strategies—prompting intensified nutrition/exercise prescriptions and closer complication surveillance—rather than as static baseline descriptors recorded once at listing.

### Immunosuppression and the metabolic-immune trade-off

Liver transplant recipients require lifelong immunosuppression to prevent rejection 10. Notably, reported rates of acute cellular rejection vary substantially across eras and definitions, with many series still citing an overall incidence in the 20-40% range—underscoring that “rejection risk” is not a binary variable but a probabilistic, time-dependent exposure 32. Reported rates of acute rejection vary across cohorts and eras; in one contemporary series, 20.5% of recipients experienced acute rejection, with most episodes occurring within the first months after LT and most responding to methylprednisolone. Even when rejection occurs, most early episodes are steroid-responsive and typically cluster within the first month, so the dominant long-run challenge becomes balancing immunosuppressive intensity against non-immunologic hazards 32. Immunosuppressive exposure nevertheless carries clinically relevant trade-offs. In contemporary cohorts, infectious events remain frequent: 45% of recipients experienced at least one infectious episode within the first 6 months post-transplant, and the burden (recurrent episodes) tracks worse long-term survival—making “infection risk” a measurable consequence of the same exposure that protects the graft 33. It increases vulnerability to infection and can contribute to long-term malignant risk, while also aggravating metabolic toxicity 4, 10, 34. This malignant liability is not trivial: *de novo* malignancy has been estimated at 10-14.6% by 5 years and 20-32% by 10 years after LT, consistent with a durable impairment of immunosurveillance under chronic immunosuppression 35. Obesity and type 2 diabetes frequently develop after LT. Meta-analytic evidence suggests that post-transplant metabolic syndrome affects 39% of recipients, and across studies it is repeatedly linked to higher cardiovascular-event incidence—meaning metabolic toxicity can translate into “hard” clinical endpoints rather than cosmetic weight change 36. Similarly, time-resolved synthesis indicates that new-onset diabetes after transplantation rises from 19.2% at 1 year to 25.8% at 5 years and 45.0% at 10 years (clear-definition sensitivity analysis), supporting the need to treat dysglycemia as a longitudinal complication rather than an early perioperative footnote 37. Clinically, early post-LT weight gain can be rapid: one longitudinal cohort reported 7.3% mean body-weight gain by 12 months with overweight/obesity prevalence increasing from 53% to 77%, and evidence of altered glucose metabolism emerging as early as 6 months—highlighting that the “metabolic phenotype” can drift quickly during recovery 38. These states sustain chronic low-grade inflammation and may modulate allograft immune responses 25, 28. Malnutrition and muscle wasting compromise host defense and increase susceptibility to infection. In this context, the liver's metabolically shaped microenvironment modulates Kupffer cell and lymphocyte function 30. Post-transplant risk assessment should therefore incorporate metabolic status, with explicit attention to muscle and nutrition, because these factors inform immunological vulnerability within the immunometabolic network. Accordingly, “adequate immunosuppression” should be framed as a calibrated target that is periodically re-anchored to contemporaneous infection burden and metabolic reserve, rather than a fixed-dose doctrine applied uniformly across heterogeneous body-composition phenotypes 33.

Collectively, this section highlights that contemporary LT outcomes are shaped by an immunometabolic risk landscape in which adverse body composition and immunosuppressive exposure converge. Sarcopenia and related phenotypes are prevalent before transplantation, often persist after LT, and are consistently associated with higher complication burden and lower survival, supporting their role as clinically actionable risk markers rather than epiphenomena. At the same time, lifelong immunosuppression imposes a necessary trade-off that can amplify metabolic toxicity and infection susceptibility, with obesity, diabetes, malnutrition, and muscle wasting each shifting immune competence and inflammatory tone. These observations support an integrated approach to post-transplant assessment that formally incorporates muscle and nutritional status alongside conventional immunologic considerations. Operationally, this argues for a dual-axis framework—immunologic risk (rejection/alloreactivity) plus immunometabolic capacity (body composition, nutrition, glycemia)—so that immunosuppressive decisions are made against a quantified host “tolerance envelope” rather than graft metrics alone 32.

## Skeletal muscle as an immunometabolic organ: substrate supply, inflammatory tone and liver-muscle signaling

### Substrate supply for immune responses

Muscle mass and immune function are closely interlinked. Muscle provides amino acids (glutamine, alanine) that fuel immune cell proliferation and the acute phase response during stress. Severe muscle wasting, as seen in sarcopenic cirrhosis patients, is associated with immune dysfunction - including fewer and less responsive T-cells and natural killer (NK) cells, and an increased risk of infections 39-42. Notably, this positions muscle depletion as an upstream constraint on host defense capacity rather than a passive correlate of advanced disease.

### Inflammation-driven catabolic amplification

Chronic inflammation, common in cirrhosis, feeds a vicious cycle wherein pro-inflammatory cytokines like TNF-α and IL-1β promote muscle protein catabolism, and the loss of muscle in turn worsens systemic inflammation 21. However, this loop is clinically consequential because it couples “immune activation” to progressive loss of physiological reserve, thereby compounding vulnerability to subsequent inflammatory hits.

### Ammonia-myostatin liver-muscle crosstalk

One striking example of liver-muscle immune crosstalk is via ammonia: in cirrhosis, hyperammonemia occurs due to reduced hepatic detoxification. Skeletal muscle helps compensate by taking up ammonia and incorporating it into glutamine; but chronically high ammonia triggers molecular changes in muscle that cause wasting. Ammonia directly upregulates myostatin (a muscle-growth inhibitor), as shown in experimental models 43. Myostatin, in turn, not only causes muscle atrophy but can also induce insulin resistance in skeletal muscle and liver 44, 45. Thus, the failing liver's inability to clear ammonia leads to a toxic signal that mediates muscle loss (*via* myostatin) - a clear demonstration of an immune-metabolic crosstalk between liver and muscle 43-45. Notably, hyperammonemia induces NF-κB-dependent transcriptional up-regulation of myostatin in skeletal muscle, providing a mechanistic anchor for this axis 43.

Therefore, hyperammonemia should be treated as an actionable upstream driver when interpreting sarcopenia trajectories in cirrhosis and the transplant pathway, rather than as a background biochemical abnormality.

### Immune-cell infiltration within skeletal muscle

Furthermore, muscle itself hosts immune cells (such as macrophages and T-cells) especially in inflammatory or atrophic conditions. In conditions like cachexia or sarcopenia, muscle-infiltrating immune cells produce cytokines (e.g. muscle TNF and IL-6) that exacerbate proteolysis 46, 47. In these cases, skeletal muscle becomes a site of cytokine production that can amplify systemic inflammatory tone and accelerate proteolytic signaling.

### Exercise-induced anti-inflammatory myokines

Conversely, in physiologic states like exercise, muscle releases anti-inflammatory myokines (e.g. IL-6, IL-10, decorin) that can dampen systemic inflammation 8. Notably, during exercise, muscle-derived IL-6 can stimulate circulating anti-inflammatory cytokines (including IL-10 and IL-1ra) and suppress TNF-α production, supporting a coherent anti-inflammatory mechanism for structured physical activity 48.

### Synthesis and transplant relevance

Taken together, skeletal muscle is a key regulator of immune-metabolic homeostasis: it disposes of glucose and lipids, buffers toxins (like ammonia), and communicates with the liver and immune system through myokines and other mediators 39, 42, 49. All these roles are highly relevant in the transplant setting, where metabolic derangements and immune activation must be carefully balanced. Collectively, this argues for embedding muscle status (quantity, quality, and function) and key metabolic toxins (e.g. ammonia) into a routine transplant-facing assessment framework, with predefined reassessment timing and escalation triggers.

## Muscle-liver immunometabolic signaling: integrating nutrient sensing, myokines and inflammatory mediators

### AMPK-mTOR nutrient sensing and muscle-to-liver metabolic relay

The broader nutrient-sensing and inflammatory circuitry linking skeletal muscle and liver in the post-transplant setting is outlined in Figure 1. Key myokines implicated in muscle-liver crosstalk and their principal metabolic and immune effects are summarized in Table 1. Beyond secreted cytokines, muscle and liver are connected by shared metabolic pathways and sensors 50. Two master regulators are AMP-activated protein kinase (AMPK) and mechanistic target of rapamycin (mTOR), which have opposing actions 50. AMPK is activated in muscle during energy stress (exercise or fasting) and triggers catabolic, energy-generating processes - e.g. glucose uptake, β-oxidation - while shutting down anabolic pathways 51. When muscle AMPK is activated (say, by exercise or pharmacologically by metformin), it not only improves muscle's own insulin sensitivity but also reduces glucose output by the liver and improves hepatic insulin sensitivity (partly through lower circulating glucose/lipid levels and possibly via myokines like BAIBA) 50. *β-Amino-isobutyric acid (BAIBA)* is a small molecule myokine released in an AMPK/PGC1α-dependent manner; BAIBA has been shown to induce hepatic fatty acid oxidation and reduce lipogenesis via PPARα activation 52. This exemplifies how muscle's energetic state can influence liver metabolism through metabolite signals. In contrast, mTOR is a nutrient-sensing kinase that promotes protein and lipid synthesis when nutrients are abundant. High mTOR activity (as seen in overnutrition or in states of excess insulin/IGF-1) in skeletal muscle can contribute to anabolic resistance and impaired insulin signaling 50. From a liver perspective, systemic activation of mTOR (for example, due to high amino acid and insulin levels) may exacerbate hepatic steatosis and reduce ketogenesis.

Notably, this AMPK-mTOR opposition provides a clinically interpretable “directional axis”: energy-stress signaling tends to improve systemic insulin sensitivity, whereas nutrient-excess signaling can entrench anabolic resistance and steatosis.

### mTOR inhibition in transplantation and downstream metabolic-muscle trade-offs

Interestingly, in transplantation, we often deliberately inhibit mTOR with drugs like rapamycin (to prevent organ rejection). This has metabolic consequences: mTOR inhibitors tend to worsen the lipid profile (causing hypertriglyceridemia and accumulation of free fatty acids, as the liver ramps up lipogenesis when mTOR's feedback on SREBP is removed) 53, 54. They may also impair muscle hypertrophy, since mTOR signaling is essential for muscle protein synthesis - potentially limiting muscle recovery post-transplant 55, 56. The balance between AMPK and mTOR pathways in muscle could thus significantly affect a transplant patient's metabolic health. *PGC-1α (Peroxisome proliferator-activated receptor gamma coactivator 1-alpha)* is another crucial player - it is a transcriptional coactivator that drives mitochondrial biogenesis and oxidative metabolism in both muscle and liver. In skeletal muscle, PGC-1α is induced by endurance exercise and mediates many benefits of training (e.g. increased oxidative fibers, angiogenesis, and myokine secretion such as irisin) 57. Muscle-specific PGC-1α has been implicated in inter-organ crosstalk: one study showed that mice with high muscle PGC-1α had increased expression of certain liver detox genes and an anti-inflammatory profile 58, whereas low PGC-1α was associated with more inflammation in both muscle and liver. Although not fully elucidated, it seems that muscle PGC-1α might send “exercise signals” that improve liver function (possibly via myokines or metabolites). Notably, this positions PGC-1α less as a muscle-only mitochondrial regulator and more as a “system-level coordinator” of oxidative capacity and inflammatory tone across organs.

Conversely, PGC-1α in the liver itself is important for gluconeogenesis and fatty acid oxidation; in insulin-resistant states, hepatic PGC-1α can be dysregulated 59. Thus, therapies that activate PGC-1α (like exercise, or potentially certain agonists) may confer systemic benefits 51. In sum, metabolic regulators like AMPK/mTOR and PGC-1α form a network whereby muscle activity and nutrient status influence liver metabolism. Targeting these pathways in muscle (for example, through exercise mimetics or AMPK activators) could be a strategy to improve metabolic outcomes after LT.

Therefore, the translational objective is pathway rebalancing toward measurable metabolic and functional endpoints, rather than pathway activation in isolation.

### Immune mediators and bidirectional inflammatory signaling

Immune Mediators and Inflammatory Crosstalk: The muscle-liver axis also involves direct immune signaling. Chronic liver disease and transplantation involve persistent immune activation - a milieu of cytokines, danger signals, and immune cell infiltration. Muscle can modulate this immune tone. For instance, muscle-derived IL-6 (again) has an immune role: during exercise, IL-6 from muscle acts on immune cells to suppress TNF-α production and stimulate anti-inflammatory IL-10, contributing to a systemic anti-inflammatory effect. This is one reason exercise is known to reduce chronic low-grade inflammation. In a post-transplant patient, regular physical activity could thus help counteract the pro-inflammatory tendencies that contribute to atherosclerosis or even allograft inflammation. On the flip side, muscle wasting often coexists with elevated circulating inflammatory cytokines (like TNF and IL-1). These cytokines can originate from the liver's Kupffer cells or from adipose tissue and directly cause muscle proteolysis via NF-κB and ubiquitin-proteasome activation 60. The liver transplant setting is unique: during the transplant surgery and early post-transplant, there is an intense inflammatory surge (ischemia-reperfusion injury in the graft, surgical stress response) 61. Patients with more robust muscle might weather this “cytokine storm” better by providing substrates for acute phase responses and by producing immunoregulatory myokines.

This section implies a time-critical window: perioperative inflammatory load interacts with baseline muscle reserve to shape early vulnerability, supporting structured assessment rather than passive observation.

### Endocrine and chemokine circuits: FSTL1 and hepatokines including FGF21

Another intriguing aspect of immune crosstalk is the role of *follistatin-like protein 1 (FSTL1)* recently identified. FSTL1 is a secreted glycoprotein that can act on immune cells; it was found to be part of a muscle-to-liver signaling pathway in NASH. Specifically, skeletal muscle IRF4 (a transcription factor) induces FSTL1 expression in muscle, which then circulates to the liver and promotes inflammation and fibrosis 62. Mice lacking IRF4 in muscle showed dramatically reduced hepatic inflammation/fibrosis on a NASH diet, whereas reintroducing FSTL1 erased this protection 62. Mechanistically, FSTL1 from muscle appears to activate liver macrophages (Kupffer cells) via receptors like DIP2A and TLR4/CD14, driving them toward a pro-inflammatory state 62. While this finding is in the context of fatty liver disease, it has implications for transplant immunobiology: it means that pathological signals from muscle (perhaps related to insulin resistance or adiposity, which increase IRF4-FSTL1) can worsen liver inflammation. In a transplant patient with metabolic syndrome and sarcopenic obesity, elevated muscle IRF4 and FSTL1 could hypothetically contribute to graft inflammation or accelerated fibrosis (especially in recurrent NASH). More research is needed in transplant models, but it underscores that immune mediators are part of muscle-liver communication. Muscle also produces chemokines (like IL-8 and CX3CL1) that might recruit immune cells systemically. Conversely, the liver secretes factors (“hepatokines”) such as FGF21, hepassocin, or fetuin-A that affect muscle's immunometabolic state. FGF21, for example, is a hepatokine induced by fasting; it increases energy expenditure and has been shown to improve graft survival in experimental LT by modulating immunometabolic homeostasis 63, 64. In summary, the muscle-liver axis operates at the intersection of metabolism and immunity: muscle-secreted factors can influence hepatic inflammation (e.g. IL-6, FSTL1, decorin), and liver-derived factors or systemic inflammation can influence muscle mass and function (e.g. TNF-α, ammonia, FGF21). Understanding these pathways provides a mechanistic basis for interventions to break the cycle of sarcopenia and poor outcomes in liver transplant patients 62. Collectively, these observations support a transplant-facing framework in which nutrient-sensing pathways and inflammatory mediators are considered coupled and potentially modifiable determinants of post-transplant immunometabolic homeostasis, as summarized in Figure 1 and Table 1.

## Impact of Immunosuppressants on the Muscle-Liver Axis

### Immunosuppression as indispensable exposure with predictable immunometabolic costs

Immunosuppressive medications are indispensable for graft survival, yet they often come at the cost of metabolic derangements and direct effects on muscle tissue. Understanding how these drugs influence the muscle-liver axis can guide strategies to mitigate side effects 65. The clinical challenge is not whether to immunosuppress, but how to titrate “rejection control” against quantifiable downstream liabilities in glycemia, lipids, and muscle reserve over time.

### Calcineurin inhibitors: diabetogenicity and insulin-resistance tilting of the axis

Tacrolimus and Cyclosporine. CNIs are the backbone of most immunosuppressive regimens, but they are well known to cause metabolic complications. Both tacrolimus (TAC) and cyclosporine (CsA) are diabetogenic. They induce pancreatic β-cell toxicity and reduce insulin secretion, while also causing peripheral insulin resistance. Clinically, new-onset diabetes after transplant (NODAT) occurs in a significant fraction of patients on CNIs. Tacrolimus tends to be more diabetogenic than cyclosporine 66, 67. Mechanistically, CNIs affect muscle by impairing insulin's ability to stimulate glucose uptake (by interfering with insulin signaling pathways in muscle) and possibly by reducing muscle mitochondrial function. Additionally, CNIs can cause hypertension and renal impairment; chronic tacrolimus use has been associated with muscle cramping and neurotoxic effects like tremors, suggesting it may indirectly reduce physical activity and muscle use 68-70. Regarding direct muscle mass effects: CNIs are not classically myotoxic in the way steroids are, but prolonged use might contribute to a sarcopenic phenotype via reduced exercise tolerance and metabolic side effects. On the liver side, CNIs modestly increase the risk of *de novo* NAFLD by promoting post-transplant diabetes and dyslipidemia. Cyclosporine, for example, can raise triglyceride and LDL levels, partly by interfering with bile acid metabolism (inhibiting hepatic 26-hydroxylase) 71. The muscle-liver axis under CNIs is thus tilted toward insulin resistance: muscle is less able to absorb glucose, leading to higher blood glucose that burdens the liver, and the liver secretes more VLDL (triglyceride) which can accumulate in muscle as intramyocellular fat (reducing muscle function) 54, 65.

Notably, a large time-based meta-analysis (28 studies; pooled n≈71,257) estimated NODAT incidence after LT at 15.51% (3 months), 16.09% (6 months), 18.30% (1 year), 20.86% (3 years), and 25.05% (10 years), with sensitivity analysis (clear NODAT definitions) rising to 44.95% at 10 years—supporting that “CNI diabetogenicity” is a longitudinal liability rather than an early transient event.

Conversely, in RCT-based meta-analysis in adult primary LT recipients, ciclosporin showed lower NODAT risk than tacrolimus at 12 months (RR with ciclosporin vs tacrolimus ≈0.60), providing trial-level support for the statement that tacrolimus is more diabetogenic 66.

### mTOR inhibitors: immunologic strategy with dyslipidemia and constrained muscle anabolism

Sirolimus (Rapamycin) and Everolimus. These agents inhibit the mTOR pathway, a key regulator of cell growth and metabolism. mTOR inhibitors have a mixed impact on the muscle-liver axis. On one hand, by inhibiting mTOR, they potentially limit muscle protein synthesis and adaptation (since mTOR is required for muscle hypertrophy). Patients on sirolimus have reported muscle weakness or delayed recovery of strength, though frank myopathy is less common than with steroids 55. On the other hand, mTOR inhibitors might *protect* muscle in certain ways: mTOR is implicated in age-related sarcopenia and metabolic syndrome, and low-dose rapamycin has been shown in some studies to improve longevity and metabolic profiles in animal models. However, in transplant patients, the dominant observed effects are negative on metabolism. Sirolimus is notorious for causing dyslipidemia - often more severe than CNIs cause 72, 73. It increases triglycerides, LDL, and total cholesterol significantly. The mechanism involves increased hepatic lipogenesis (rapamycin paradoxically boosts SREBP-driven lipid synthesis) and reduced lipid clearance. Hypertriglyceridemia from sirolimus can lead to ectopic fat deposition in muscle (myosteatosis) and liver (steatosis). Additionally, some evidence suggests rapamycin may reduce muscle endurance by affecting mitochondrial oxidative capacity. In terms of direct muscle-liver signaling, mTOR inhibitors could theoretically alter myokine production (since mTORC1 influences protein translation, including that of cytokines). For example, inhibition of mTOR in muscle might blunt the exercise-induced rise in beneficial myokines like irisin, though this has not been conclusively shown in humans. Clinically, patients switched from a CNI to an mTOR inhibitor often experience weight gain if appetite increases (because sirolimus does not cause the same appetite suppression that tacrolimus sometimes does due to GI side effects). Overall, the immunosuppressive mTOR blockade may hamper muscle growth and promote a pro-steatotic metabolic profile, underscoring the need to monitor lipids and muscle strength in patients on these drugs 65.

Notably, in one liver-transplant experience of rapamycin-based primary immunosuppression, hypercholesterolemia was reported in up to 44% of patients, indicating that dyslipidemia is common enough to warrant proactive surveillance rather than reactive management 72.

Similarly, in that same experience, hypertriglyceridemia (e.g., TG > 300 mg/dL) was observed in a meaningful subset (10/57; 18%), consistent with the “ectopic fat deposition” narrative (muscle and liver) in susceptible recipients.

### Antimetabolites and newer agents: low direct metabolic toxicity but indirect nutritional-muscle exposure

Mycophenolate and Others: Mycophenolate mofetil (MMF) and azathioprine are antimetabolite immunosuppressants that primarily affect DNA synthesis in lymphocytes. They do not have prominent direct metabolic side effects like CNIs or steroids. However, MMF can cause gastrointestinal side effects (nausea, malabsorption, diarrhea) which, if severe, might contribute to malnutrition and muscle wasting indirectly 74. Patients with chronic diarrhea from MMF may lose weight and muscle mass; dose reduction or switching to enteric-coated formulations can help 75. Azathioprine's effects on muscle/liver axis are minimal, though it can cause mild insulin resistance. Newer agents like IL-2 receptor blockers or co-stimulation blockers (belatacept) have little known impact on muscle or metabolism, aside from allowing lower doses of steroids or CNIs which indirectly benefits metabolic outcomes 65.

Accordingly, any metabolic benefit attributed to these agents in LT should be interpreted mainly in the context of steroid minimization or calcineurin-inhibitor sparing, rather than as a direct drug-specific effect; where LT-specific evidence is unavailable, this should be stated clearly, and findings from other solid-organ transplant settings should not be generalized uncritically.

### Interactions and emerging insights: additive toxicity and microbiome-linked muscle loss

It is important to recognize that immunosuppressant effects on muscle-liver axis can be synergistic or additive. For example, a patient on tacrolimus *and* high-dose steroids is at very high risk for rapid muscle atrophy and metabolic syndrome due to the combined diabetogenic and protein-catabolic effects. By contrast, centers that minimize steroids and aim for lower tacrolimus troughs often report patients have better weight control and muscle function post-transplant 65. Emerging evidence from murine models and kidney-transplant cohorts implicates the gut microbiota as a potential intermediary linking immunosuppressive exposure to skeletal-muscle loss 76. In a 2025 study integrating mouse experiments with observational data from kidney transplant recipients, immunosuppressant-associated microbiota perturbation was linked to reduced muscle mass, pointing to a previously underappreciated gut-muscle dimension of post-transplant catabolism. Calcineurin inhibitors and corticosteroid-containing regimens may contribute to dysbiosis-related alterations in this axis, although the underlying mechanisms and their relevance to liver transplantation remain undefined. These data should therefore be regarded as hypothesis-generating rather than LT-specific evidence. Nevertheless, they raise the possibility that microbiota-directed interventions may eventually help attenuate selected immunosuppression-related muscle effects, a concept that now requires direct validation in LT cohorts 76. Additionally, some immunosuppressants have direct neuromuscular side effects (tacrolimus can cause neuropathy or motor neuron toxicity in high levels), which can manifest as muscle weakness. Recognizing drug-induced etiologies of muscle weakness is important so that they are not misattributed to deconditioning alone 27. In summary, while immunosuppressants are essential, they often “tip the scales” of the muscle-liver axis toward a pathological state: fostering insulin resistance, hyperlipidemia, and muscle protein loss 77.

Notably, steroid minimization has been associated with measurable reductions in metabolic side effects under tacrolimus-based regimens (e.g., lower post-transplant diabetes and hyperlipidemia rates with early steroid withdrawal), supporting that “regimen architecture” can shift the immunometabolic burden 78.

Collectively, this section justifies treating immunosuppressant selection and dosing as an upstream determinant of body-composition trajectory, with predefined monitoring targets in glycemia, lipids, and muscle function rather than relying on weight change alone.

## Mechanistic insights from models and multi-omics: decoding muscle-liver signaling circuits

### Hyperammonemia-myostatin liver-to-muscle signaling

A seminal animal study demonstrated how a failing liver can induce muscle wasting via a specific molecular mechanism. In a chronic liver injury model, researchers showed that elevated ammonia levels led to increased myostatin expression in skeletal muscle, which in turn caused sarcopenia 43. Notably, hyperammonemia stimulated myostatin transcription in an NF-κB-dependent manner in muscle cells, providing a defined mechanistic link between ammonia burden and muscle catabolism 43. Ammonia was thus identified as a hepatogen (liver-derived factor) driving muscle loss - a prime example of liver-to-muscle crosstalk 43. Importantly, treating hyperammonemia (for instance with ammonia-lowering therapies like L-ornithine L-aspartate or rifaximin) attenuated muscle wasting in these models. In portocaval anastomosis rats, 4 weeks of oral ammonia-lowering therapy with L-ornithine L-aspartate plus rifaximin significantly lowered blood and skeletal muscle ammonia and increased lean body mass and grip strength, with reversal of myostatin/autophagy perturbations 79. This finding has direct implications for cirrhotic patients awaiting transplant: aggressively managing ammonia (to prevent hepatic encephalopathy) might also preserve muscle mass by breaking the ammonia-myostatin-sarcopenia cycle 43. After transplant, ammonia typically normalizes, but some patients (e.g. with small-for-size grafts or transient dysfunction) might have elevated ammonia which could harm newly regenerating muscle - a consideration for post-transplant care.

### IRF4-FSTL1 muscle-to-liver endocrine pathway in steatohepatitis models

Discussed earlier, the Nat Commun (2023) study by Guo *et al.* used muscle-specific IRF4 knockout mice to probe muscle→liver signals in fatty liver disease. They found muscle IRF4 deletion protected mice from NASH (less steatohepatitis and fibrosis) without affecting body weight. Proteomic analysis pointed to lower FSTL1 as the key change, and experiments restoring FSTL1 in those mice brought back the liver damage. Furthermore, co-culture of liver cells with FSTL1 confirmed its direct effects: it activated inflammatory pathways in Kupffer cells and pro-fibrogenic pathways in stellate cells via distinct receptors. Notably, this work mapped a muscle IRF4-FSTL1-DIP2A/CD14 signaling axis and reported a positive correlation between serum FSTL1 and human NASH progression. These findings identify a muscle-to-liver endocrine pathway that may also be relevant in humans. Indeed, the authors found serum FSTL1 levels in patients correlated with NASH severity. In transplantation, this pathway may be particularly relevant to recurrent NASH and other metabolically vulnerable graft-injury phenotypes. One scenario is recurrence of NASH after transplant: muscle IRF4 might be highly expressed in patients with insulin resistance or obesity, leading to more FSTL1 and a higher risk of graft NASH. Therapies that block FSTL1 or modulate muscle IRF4 (perhaps indirectly via exercise or PPARδ agonists, since IRF4 in muscle is involved in oxidative metabolism) could potentially protect the graft. While speculative at this stage, it exemplifies how animal studies guide us to novel targets along the muscle-liver axis.

### Multi-omics as a bridge from mechanism to actionable nodes

In summary, animal and laboratory studies reinforce the concept that muscle and liver communicate via specific molecules (like myostatin, FSTL1, CHI3L1) and that disturbing this communication in one organ can have major effects on the other. Notably, a complementary multiomics study integrating transcriptomics with mitochondrial proteomics in hyperammonemic myotubes and portocaval anastomosis rats identified ETC remodeling and a senescence-associated signature that was partially reversible with ammonia withdrawal/lowering 80. These insights not only support the clinical relevance of muscle-related phenotypes for post-transplant outcomes, but also point to concrete targets for intervention - for example, blocking myostatin to prevent sarcopenia, or inhibiting a muscle-derived inflammatory myokine to reduce liver fibrosis. As we move forward, integrating omics data from both muscle and liver of transplant patients could identify biomarkers to predict who is at risk of frailty or metabolic complications, enabling personalized therapy to modulate the muscle-liver axis 43. At present, translation of these omics-defined nodes to liver transplantation requires longitudinal paired sampling and standardized phenotyping, both of which remain limited in clinical cohorts; the dynamic remodelling of the liver-muscle axis across the pre-LT, peri-LT and post-LT continuum is summarized in Figure 2.

## Therapeutic leverage points along the muscle-liver immunometabolic axis: from rehabilitation to precision strategies

**Figure 3:**
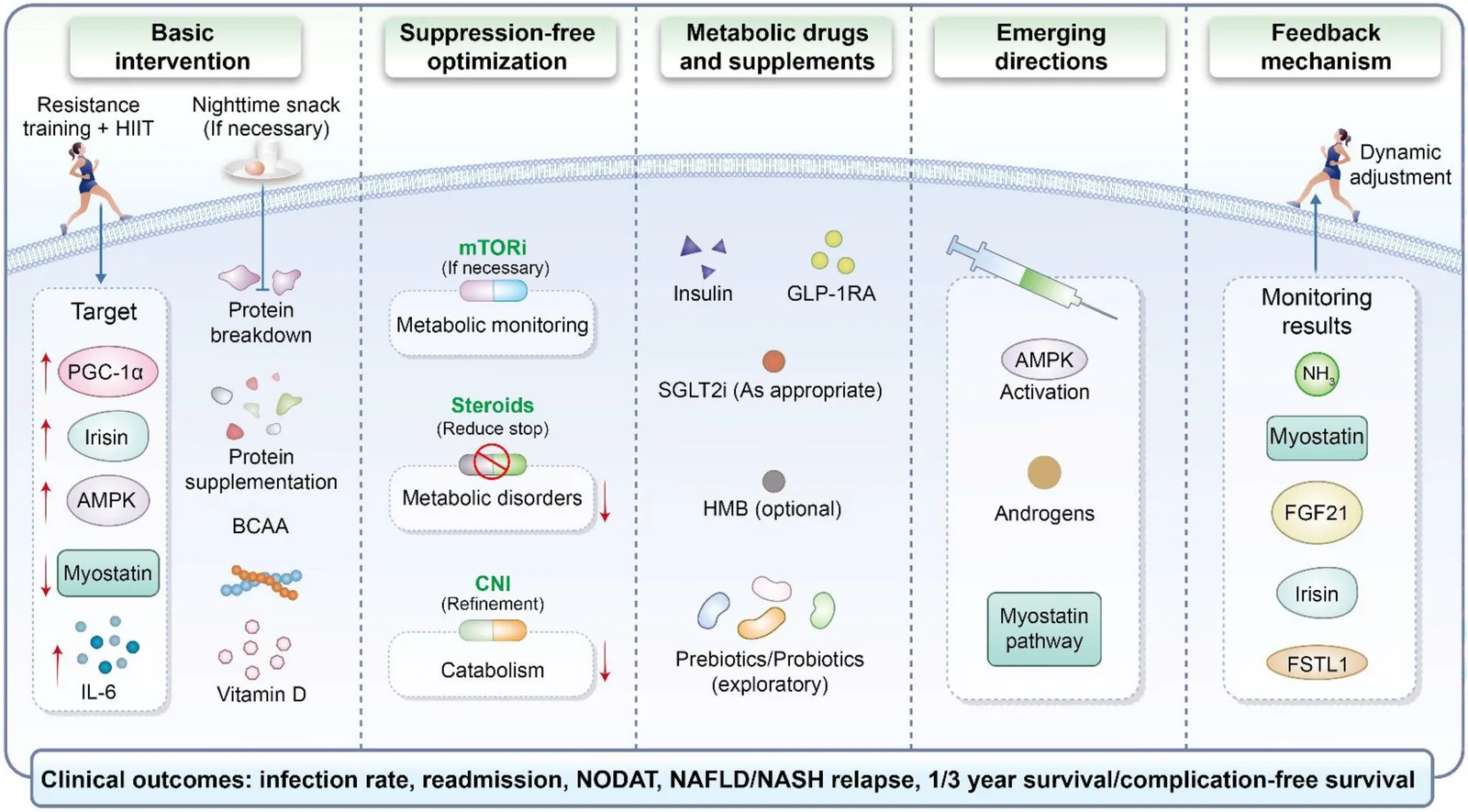
** A translational management framework targeting the post-transplant muscle-liver immunometabolic axis.** This schematic depicts an integrated management framework comprising basic intervention, immunosuppression-related optimization, metabolic drugs and supplements, emerging therapeutic directions, and a feedback mechanism for monitoring results and dynamic adjustment. The illustrated interventions include exercise training, nutritional support, individualized modification of immunosuppressive exposure, and selected metabolic or pathway-targeted approaches. Potential targets shown in the figure include PGC-1α, irisin, AMPK, myostatin, and IL-6, whereas the proposed monitoring indicators include ammonia, myostatin, FGF21, irisin, and FSTL1. The clinical outcomes of interest shown at the bottom of the schematic include infection rate, readmission, new-onset diabetes after transplantation, NAFLD/NASH relapse, and 1-/3-year survival or complication-free survival.

Appreciating the muscle-liver immunometabolic axis in liver transplantation suggests that interventions aimed at muscle could have profound benefits for transplant patients. A multidimensional strategy - combining exercise, nutritional support, and possibly pharmacological agents - is likely needed to address sarcopenia and metabolic dysfunction in this population. A clinically oriented management framework for targeting the post-transplant muscle-liver immunometabolic axis is outlined in Figure 3. As summarized in Figure 3, these approaches span foundational lifestyle measures, optimization of immunosuppressive exposure, adjunctive metabolic therapies and emerging pathway-directed strategies. Notably, major liver-disease nutrition guidance already frames sarcopenia care around “training + adequate protein”, providing an explicit, implementable anchor for LT pathway design.

### Exercise-based prehabilitation and structured rehabilitation

Exercise is a cornerstone therapy to enhance the muscle-liver axis. Numerous studies have demonstrated that supervised exercise programs for cirrhotic patients awaiting LT are safe and improve fitness and muscle mass 81-83. Prehabilitation (pre-transplant exercise training) has been associated with better functional capacity and possibly fewer post-transplant complications. For example, a home-based moderate exercise program in LT candidates improved their 6-minute walk distance and was well-tolerated, even in those with ascites and fatigue. In a home-based prehabilitation pilot in LT candidates, Liver Frailty Index improved from 3.84 ± 0.71 to 3.47 ± 0.90 and 6-minute walk distance increased from 318 ± 73 m to 358 ± 64 m, supporting near-term reversibility of functional frailty with structured training 84.

Post-transplant, exercise remains equally important. Engaging in resistance and aerobic training during the recovery phase can accelerate gains in muscle strength and help counteract the muscle atrophy that occurred during the illness and perioperative period 85, 86. One pilot study in liver transplant recipients found that a 12-week supervised exercise regimen led to improved VO₂ max, increased lean body mass, and better quality of life 85, 86. Another trial noted that patients who achieved a certain improvement in their Liver Frailty Index through exercise had better 1-year survival than those who remained frail 87. Mechanistically, exercise exerts its benefits by activating muscle hypertrophy pathways (mTOR when combined with protein nutrition), enhancing mitochondrial function (through PGC-1α), and releasing beneficial myokines (like IL-6, irisin, IL-15) that improve insulin sensitivity and reduce liver fat 88-90. It also likely suppresses harmful myokines (reducing myostatin levels, for instance) 91. For transplant programs, incorporating structured exercise - perhaps starting in the hospital early post-transplant under supervision and continuing as outpatient rehab - may yield dividends in reducing hospital readmissions and improving graft outcomes. *High-intensity interval training (HIIT)* and *resistance training* both have shown promise in other solid organ transplant patients (e.g. heart transplant), and similar approaches could be tailored for liver recipients, bearing in mind any incision or hernia concerns. In younger transplant recipients (such as pediatric cases who have grown up post-transplant), promoting lifelong physical activity is crucial to stave off metabolic syndrome and muscle loss later in life 92.

In a randomized trial after orthotopic liver transplantation, combined exercise plus dietary counseling improved physiologic and functional endpoints versus usual care (including VO₂peak, quadriceps strength, and physical function measures), underscoring that “rehabilitation dose” can be trialed as an intervention rather than a general recommendation 86.

### Protein targets, supplementation, and diet quality as anabolic and metabolic guardrails

Nutrition is the other pillar of muscle-centric therapy. Protein-calorie malnutrition is prevalent in cirrhosis and often persists in the early post-transplant period due to recovery demands and sometimes prolonged hospitalization. Guidelines recommend high protein intake (1.2-1.5 g/kg/day) for cirrhotic patients to combat sarcopenia. This should continue after transplant, as protein is needed to rebuild lost muscle. Notably, liver patients tolerate protein better once the liver is functioning (since the fear of precipitating encephalopathy with high protein largely disappears after transplant), so one can be aggressive in protein supplementation 93. Therefore, “1.2-1.5 g/kg/day” functions as a concrete prescription-level target rather than a narrative statement, and protein restriction is not routinely supported as a sarcopenia strategy in cirrhosis guidance 93.

Branched-chain amino acids (BCAAs) have a special role: BCAAs (leucine, isoleucine, valine) serve as both substrates for muscle synthesis and signals to stimulate mTOR pathway in muscle. In cirrhosis, BCAA levels are often low relative to aromatic amino acids. Multiple trials have found that oral BCAA supplementation in cirrhotic patients improves muscle mass, muscle strength, and even albumin levels 94. A meta-analysis showed that long-term BCAA supplementation improved sarcopenia and prognostic nutritional indices in advanced liver disease. Post-transplant, while formal studies are fewer, it is plausible that continued BCAA supplementation (for those with suboptimal oral intake) could support muscle anabolism during rehabilitation 95-97. In a randomized trial of frail compensated cirrhosis, oral BCAA improved Liver Frailty Index over 16 weeks compared with control (ΔLFI -0.31 vs +0.02), aligning supplementation with a measurable frailty readout rather than weight change alone 98.

Similarly, ensuring adequate vitamin D and micronutrient levels is important - vitamin D deficiency is common in liver disease and contributes to myopathy; correcting it may improve muscle function. Frequent meals or nighttime snacks are recommended for cirrhosis to avoid prolonged catabolic fasting periods. After transplant, patients often revert to a normal eating pattern, but if muscle rebuilding is a goal, nutritional strategies from sports medicine (like protein intake spread across meals, protein + carbohydrate supplement immediately after exercise to maximize muscle protein synthesis) could be employed. Dietary fat content should be watched as well: limiting simple sugars and saturated fats can help prevent excessive fat gain in liver and muscle 99. Given the high incidence of post-transplant metabolic syndrome, a Mediterranean-style diet (rich in fruits, vegetables, lean protein, and healthy fats) may both protect the graft (less steatosis) and provide ample nutrients for muscle.

However, robust post-LT randomized evidence for specific dietary patterns remains limited, so diet selection should be framed as metabolically protective and feasible rather than definitively outcome-modifying.

### Pharmacological and precision strategies: anabolic agents, hormones, and regimen selection

In addition to lifestyle, there is growing interest in drugs to specifically target muscle wasting in liver disease. One major target is the myostatin pathway. *Myostatin antagonists* (like monoclonal antibodies or receptor blockers) are being tested in various conditions of muscle loss 95. In theory, blocking myostatin in a post-transplant patient could accelerate muscle gain and strength recovery. There is precedent in other fields: a myostatin antibody (bimagrumab) increased muscle mass in clinical trials for sarcopenia and metabolic improvement, though results were mixed for functional outcomes 100, 101. In cirrhosis models, myostatin inhibition clearly improves muscle mass. Experts have posited that such agents “could potentially contribute to improvement of sarcopenia” in chronic liver disease 102. We may see trials of myostatin inhibitors in pre- or post-transplant patients in the near future. Another hormonal avenue is *androgen therapy*. Many male cirrhosis patients are hypogonadal, and low testosterone correlates strongly with sarcopenia, frailty, and mortality 103. Notably, a 12-month placebo-controlled trial of testosterone in men with cirrhosis reported gains in lean mass and reductions in fat mass with improvement in selected metabolic markers, supporting testosterone as an anabolic lever in appropriately selected hypogonadal patients 104, 105.

After transplant, testosterone levels often normalize if the liver was the cause (since sex hormone-binding globulin drops and gonadal function may recover), but some patients remain hypogonadal (due to long-standing endocrine dysfunction or immunosuppressant effects). In those cases, careful testosterone therapy might be beneficial for muscle and bone health - though it must be balanced against potential risks (polycythemia, prostate issues). In women, the role of hormone replacement (e.g. estrogen) on muscle is less clear; estrogen has some muscle benefits but also can reduce protein breakdown.

On the horizon, therapies targeting the muscle-liver axis could become part of standard transplant care. For instance, if myostatin inhibitors prove safe, they might be given to sarcopenic patients pre-transplant to build a “reserve” of muscle before surgery, or shortly after transplant to hasten rehabilitation (some analogies exist in cardiac surgery where pre-op exercise improved outcomes). Gene therapy is another distant possibility: e.g. delivering Follistatin (a natural myostatin blocker) via viral vectors to patients to boost muscle - studies in muscular dystrophy are exploring this. Moreover, the concept of *personalized immunosuppression* could extend to choosing drugs that best fit a patient's metabolic profile. At present, belatacept-based regimens cannot be regarded as standard metabolic-sparing options in *de novo* adult liver transplantation, because phase II trial data showed inferior safety/efficacy signals compared with tacrolimus-based regimens; accordingly, their applicability in LT remains highly constrained and requires explicit evidence-based justification 106.

Taken together, these strategies can be integrated into a dynamic management framework in which lifestyle measures, immunosuppressant optimization, adjunctive metabolic therapies and biomarker-guided feedback are combined to improve clinically relevant post-transplant outcomes, as summarized in Figure 3.

## Knowledge gaps and prospective studies

Despite growing recognition of the muscle-liver axis in liver transplantation, clinical translation remains constrained by non-standardized phenotyping, limited mechanistic and longitudinal evidence, and insufficient interventional data 107, 108. Substantial heterogeneity in imaging assessment of muscle mass and muscle quality, diagnostic thresholds, contrast phase, and assessment timing continues to weaken comparability across studies, while conventional anthropometric measures are often confounded by ascites, edema, and peri-transplant fluid shifts, reducing sensitivity for clinically relevant phenotypes such as myosteatosis, sarcopenic obesity, and impaired functional reserve 107, 109. At the same time, the predominance of retrospective and associative data leaves the temporal and causal significance of muscle abnormalities incompletely defined 107. Future research should therefore focus on standardized longitudinal phenotyping from wait-listing to post-transplant recovery, biomarker-integrated mechanistic studies, and transplant-specific endpoints that account for competing risks 107, 108. In parallel, adequately powered multicenter prospective trials are required to determine whether prehabilitation, structured rehabilitation, and individualized nutritional support can deliver clinically meaningful benefit 110.

## Conclusion

Taken together, current evidence supports a clinically important role for the muscle-liver immunometabolic axis in liver transplantation 7, 65. In the LT setting, skeletal muscle should be regarded not merely as a marker of nutritional status, but as an active component of metabolic and inflammatory regulation 6, 8. Sarcopenia and frailty are consistently associated with adverse waitlist and post-transplant outcomes, while myosteatosis and sarcopenic obesity provide additional prognostic information after LT 19, 20, 26. Mechanistically, this bidirectional axis appears to involve myokine-mediated signaling, hyperammonemia-driven muscle catabolism, and liver-derived inflammatory and metabolic stress pathways 43, 62. In parallel, immunosuppressive exposure may further aggravate glucose and lipid dysregulation and contribute to skeletal muscle loss 65, 76.

From a clinical perspective, these observations support closer integration of body-composition phenotyping and frailty assessment into LT care pathways, together with structured peri-transplant rehabilitation 107, 111. However, much of the current evidence remains observational, and LT-specific longitudinal data are still limited 19, 107. Further transplant-specific prospective and interventional studies are needed to determine whether prehabilitation, post-transplant rehabilitation, and nutritional optimization can improve clinically meaningful outcomes after LT 86.

## Supplementary Material

Supplementary table.

## Figures and Tables

**Table 1:** Summary of Key Myokines Implicated in Muscle-Liver Crosstalk.

Myokine	Major Source & Stimulus	Effects on Liver Metabolism	Immune/Inflammatory Effects	Representative references
IL-6	Skeletal muscle (especially during exercise)	Enhances hepatic fatty acid oxidation, improves insulin sensitivity	Promotes anti-inflammatory milieu acutely (induces IL-10, suppresses TNF)	6, 48
Myostatin	Skeletal muscle (inactivity and chronic disease ↑)	Increases hepatic lipogenesis and triglyceride accumulation; suppresses fatty acid β-oxidation	Pro-atrophy (causes muscle wasting); elevated in chronic inflammation (TGF-β family member) - may promote a pro-fibrotic, pro-inflammatory state indirectly	43-45
IL-15	Skeletal muscle (constitutive, exercise-induced)	Modulates fat-muscle-liver axis; KO of IL-15 reduces hepatic steatosis (suggesting IL-15 normally promotes some lipid accumulation)	Supports lymphocyte (NK and T-cell) homeostasis; reduces visceral fat (an anti-obesity myokine), which indirectly lowers pro-inflammatory adipokines	112, 113
Irisin	Skeletal muscle (cleavage of FNDC5 via PGC-1α activation during exercise)	Decreases *de novo* lipogenesis in liver; stimulates fatty acid β-oxidation (via PPARα); associated with improved liver fat and glucose control	Lowers oxidative stress and inflammatory signaling in liver (reduces NF-κB, TNF-α, and ROS production in hepatocytes)	57
Follistatin-like 1 (FSTL1)	Skeletal muscle (IRF4-dependent expression, elevated in insulin resistance and NASH)	Worsens NASH-related pathology: promotes hepatic steatosis, inflammation and fibrosis (muscle IRF4→FSTL1 drives liver fat and injury)	Pro-inflammatory: activates liver Kupffer cells and stellate cells via receptors (e.g. DIP2A/CD14), enhancing inflammatory and fibrogenic responses	62

## Abbreviations

AMPK: AMP-activated protein kinase
BAIBA: β-aminoisobutyric acid
BCAA: branched-chain amino acids
CNI: calcineurin inhibitor
CsA: cyclosporine A
CX3CL1: C-X3-C motif chemokine ligand 1
DIP2A: disco-interacting protein 2 homolog A
FGF21: fibroblast growth factor 21
FNDC5: fibronectin type III domain-containing protein 5
FSTL1: follistatin-like protein 1
HIIT: high-intensity interval training
ICU: intensive care unit
IGF-1: insulin-like growth factor 1
IL-1β: interleukin 1β
IL-6: interleukin 6
IL-8: interleukin 8
IL-10: interleukin 10
IL-15: interleukin 15
IRF4: interferon regulatory factor 4
LT: liver transplantation
MMF: mycophenolate mofetil
mTOR: mechanistic target of rapamycin
NAFLD: non-alcoholic fatty liver disease
NASH: non-alcoholic steatohepatitis
NF-κB: nuclear factor κB
NK: natural killer
NODAT: new-onset diabetes after transplant
PGC-1α: peroxisome proliferator-activated receptor gamma coactivator 1 alpha
PPARα: peroxisome proliferator-activated receptor alpha
PPARδ: peroxisome proliferator-activated receptor delta
ROS: reactive oxygen species
SREBP: sterol regulatory element-binding protein
TAC: tacrolimus
TGF-β: transforming growth factor beta
TLR4: toll-like receptor 4
TNF-α: tumor necrosis factor alpha
VLDL: very-low-density lipoprotein
VO₂max: maximal oxygen uptake
