# Supplementary material for: The Muscle-Liver Immunometabolic Axis in Liver Transplantation: A Decisive Role in Outcomes and Mechanisms
Source: Int J Biol Sci. 2026 Jun 25;22(12):6483–99. doi: 10.7150/ijbs.132050 (PMC13411972; doi:10.7150/ijbs.132050)
Supplement: Supplementary file 1 — Supplementary table. [file ijbsv22p6483s1.pdf]

Supplementary Table S1.

Search terms for PubMed Search date: 2 April 2026

Search set	Search strategy	Records
**#1**	("Liver Transplantation" OR "liver transplantation" OR "liver transplant*"OR "orthotopic liver transplant" OR "hepatic transplant" OR "liver transplant recipient" OR "liver transplant candidate")	96277
**#2**	("Sarcopenia" OR "Frailty" OR "Body Composition" OR "Muscle, Skeletal" OR sarcopen OR frail OR "skeletal muscle" OR "muscle mass" OR "muscle quality" OR myosteatos OR "body composition" OR "sarcopenic obesity" OR "muscle attenuation"OR "muscle radiodensity")	552180
**#3**	("Myokines"OR "Exercise"OR "Exercise Therapy" OR "Rehabilitation"[mh] OR "Nutrition Therapy" OR "muscle liver axis" OR "liver muscle axis"OR myokine OR hepatokine OR immunometaboli OR prehabilitation OR rehabilitation OR "exercise therapy" OR "resistance training" OR "aerobic exercise" OR "nutritional support" OR "nutrition therapy" OR "branched-chain amino acid" OR BCAA)	961,642
**#4**	#1 AND #2 AND #3	203

Search terms for Web of Science Core Collection Search date: 2 April 2026

Search set	Search strategy	Records
**#1**	TS=("liver transplantation" OR "liver transplant*" OR "orthotopic liver transplant" OR "hepatic transplant*" OR "liver transplant recipient" OR "liver transplant candidate")	57693
**#2**	TS=(sarcopen OR frail OR "skeletal muscle" OR "muscle mass" OR "muscle quality" OR myosteatos OR "body composition" OR "sarcopenic obesity" OR "muscle attenuation" OR "muscle radiodensity")	204308
**#3**	TS=("muscle-liver axis" OR "muscle liver axis" OR "liver-muscle axis" OR "liver muscle axis" OR myokine* OR hepatokine OR immunometaboli OR prehabilitation OR rehabilitation OR "exercise therapy" OR "resistance training" OR "aerobic exercise" OR "nutritional support" OR "nutrition therapy" OR "branched-chain amino acid" OR BCAA)	214622
**#4**	#1 AND #2 AND #3	220
